# Acceptance of Assistive Technology by Users with Motor Disabilities Due to Spinal Cord or Acquired Brain Injuries: A Systematic Review

**DOI:** 10.3390/jcm12082962

**Published:** 2023-04-19

**Authors:** Sara Ventura, Giovanni Ottoboni, Alessandro Pappadà, Alessia Tessari

**Affiliations:** 1 Department of Psychology “Renzo Canestrari”, Alma Mater Studiorum—University of Bologna, Viale Berti Pichat 5, 40127 Bologna, Italy; sara.ventura7@unibo.it (S.V.); alessia.tessari@unibo.it (A.T.); 2 Instituto Polibienestar, University of Valencia, Avd. Blasco Ibañez 21, 46010 Valencia, Spain; 3 Alma Mater Research Institute for Human-Centered Artificial Intelligence, University of Bologna, 40121 Bologna, Italy

**Keywords:** assistive technology, technology acceptance, acquired neurological lesion, systematic review

## Abstract

Acquired motor limits can be provoked by neurological lesions. Independently of the aetiologies, the lesions require patients to develop new coping strategies and adapt to the changed motor functionalities. In all of these occasions, what is defined as an assistive technology (AT) may represent a promising solution. The present work is a systematic review of the scientific AT-related literature published in the PubMed, Cinahl, and Psychinfo databases up to September 2022. This review was undertaken to summarise how the acceptance of AT is assessed in people with motor deficits due to neurological lesions. We review papers that (1) dealt with adults (≥18 years old) with motor deficits due to spinal cord or acquired brain injuries and (2) concerned user acceptance of hard AT. A total of 615 studies emerged, and 18 articles were reviewed according to the criteria. The constructs used to assess users’ acceptance mainly entail people’s satisfaction, ease of use, safety and comfort. Moreover, the acceptance constructs varied as a function of participants’ injury severity. Despite the heterogeneity, acceptability was mainly ascertained through pilot and usability studies in laboratory settings. Furthermore, ad-hoc questionnaires and qualitative methods were preferred to unstandardized protocols of measurement. This review highlights the way in which people living with acquired motor limits greatly appreciate ATs. On the other hand, methodological heterogeneity indicates that evaluation protocols should be systematized and finely tuned.

## 1. Introduction

According to the World Report on Disability [1], annually, over 150 million people across the globe suffer a neurological lesion linked to acquired brain or spinal cord injury. The outcome of these events, regarding various motor deficits affecting the upper or lower extremities, vary according to the sites and the gravity of the lesions [2]. Once diagnosis and prognosis are concluded, equity must be guaranteed to any person living with a disability [3]. After discharge from the hospital, people with motor disabilities must develop new coping strategies to respond to their new bodily and motor functionalities. Indeed, they cannot complete a range of activities and require various forms of support and assistance in completing daily tasks. Such situations negatively impact social relationships, educational gains, employability, and quality of life [4].

Recently, assistive technology (AT), or “any product or technology-based service that enables people of all ages with activity limitations in their daily life, education, work or leisure” (Association for the Advancement of Assistive Technology in Europe [5], has begun to represent a promising solution in support of the fulfilment of personal needs independently of problem nosography or aetiology. ATs can be distinguished into hard and soft technologies according to their characteristics. Hard technologies are tangible devices allowing people with motor deficits to move (e.g., limb exoskeleton or wheelchair) or to act on objects (e.g., robotic arm). Hard technologies also include assistance with pointing devices, switches, and joystick control systems that could be assembled in more sophisticated ATs with the aim of supporting and enhancing motor activities (an example is the mouth or chin stick for people with quadriplegia to drive their wheelchair [6]. On the other hand, soft technologies include non-tangible devices supporting people in decision-making, strategy development, training, and concept formation. They may be available in one of three forms: people (e.g., teacher or therapist), texts (e.g., instruction manual), or computers (e.g., mixed reality and exergames) [7]. Scientific evidence from clinical trials and systematic reviews supports the efficacy of ATs in reducing impairments, improving motor functions, and enhancing social interactions, significantly benefiting patients’ and caregivers’ quality of life [8].

Considering the significant importance of ATs, it is worth developing the technology from a starting point of the patient’s point of view to ensure its acceptance [9,10,11]. The World Health Organization recently stated that, when developing an AT, it is necessary to shift from a “design for the users” approach to a “design with the users” approach. According to this, previous literature has proved that ATs suffer from an almost 75% dropout rate among users [8], and the causes are mainly due to the lack of user and caregiver involvement [11]. In this light, several theoretical models, including the technology acceptance model (TAM; [12]), the human activity assistive technology (HAAT; [13]), the matching person and technology model (MPT; [14]), and the Almere model [15]—which orients the present review—have either investigated or explained the role of technology acceptance.

Given the importance of ATs for people living with motor impairments in the improvement of their quality of life, and alongside the critical role of the acceptance to use the ATs over time effectively, the current review investigates the levels of AT acceptance of hard ATs by patients living with motor limits due to spinal cord or acquired brain injuries.

## 2. Methods

The current systematic review followed the Preferred Reporting Items for Systematic Reviews and Meta-Analyses (PRISMA) statement [16].

### 2.1. Study Selection Criteria

The inclusion criteria for the studies in this review were based on the participant, intervention, comparison, outcome (PICO) format of study design questions [17].

To be included in the systematic review, studies had to fulfil the following criteria: (a) to have a sample of participants ≥ 18 years old with—at least—a motor deficit due to a lesion secondary to the spinal cord and with acquired brain injuries but preserved cognitive functions so as to be able to lead the study; (b) to examine the patients’ acceptance about hard assistive technology; and (c) to be written in English or Italian.

### 2.2. Search Strategy

As suggested by [18], the systematic literature search was conducted on the PubMed, Cinahl, and Psychinfo databases up to September 2022. The keywords were grouped in the following three areas: (1) hardware: exoskeleton robot, assistive technology, assistive device, robot-assisted, assistive robot; (2) technology acceptance: acceptance, tam, attitude; and (3) clinical area: stroke, brain injury, brain lesion, spinal injury, spinal cord injury, paraplegic, quadriplegic. These keywords were used in combinations as follows: ((exoskeleton robot* OR assistive technolog* OR assistive devic* OR robot assisted OR assistive robot* OR neuroprost*) AND (accepta* OR “tam” OR attitude)) AND (stroke OR brain injur* OR brain lesion OR spinal injur* OR spinal cord injury OR paraplegi* OR quadriplegi*).

### 2.3. Search Outcome

**Figure 1:**
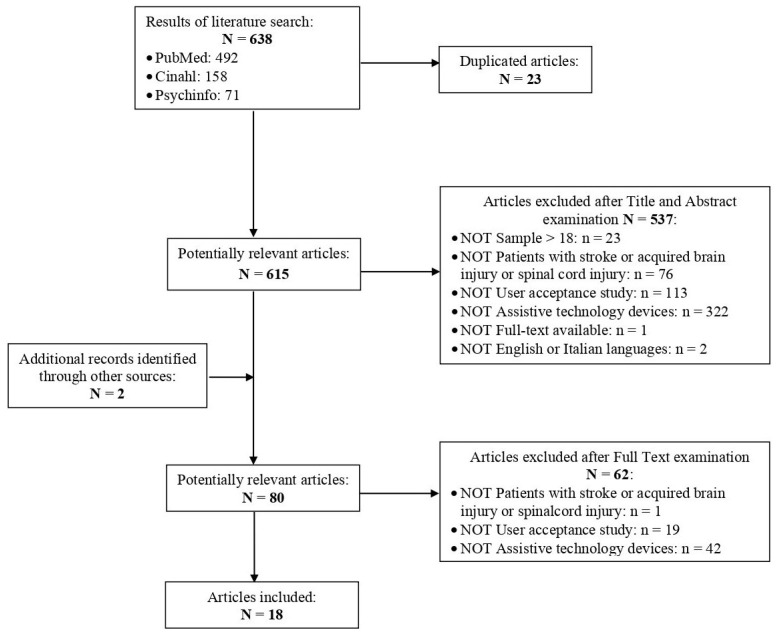
PRISMA flow diagram: selection process of studies included in the systematic review.

The initial search procedure led to 638 potentially relevant studies. After removing 23 duplicates, 615 titles and abstracts were read. Of those, 537 were removed because they did not match the focus of our research. The resulting 78 items were thoroughly read. Two further papers were discovered through the papers’ bibliographies and consequently added. Out of these 80 articles, 62 studies were excluded because they fell outside of the following inclusion criteria: they did not examine patients with acquired brain injury (*n* = 1), they did not study user acceptance (*n* = 19), and they did not study hard assistive technology (*n* = 42). At the end of the literature search process, 18 studies were selected (Figure 1). Two researchers (S.V. and A.P.) operated the selection while supervised by two authors (G.O. and A.T.).

### 2.4. Methodological Research Quality Assessment

The standard quality assessment criteria checklist was used to appraise the methodological quality of the included studies as it can guide the evaluation of health technology assessment and can check the research design and methodology, participant selection, data collection and analysis, and the statement of findings [19]. The methodological assessment tool contains 14 items for assessing the quality of quantitative studies, each of which is scored as positive (yes = 2), partial (=1), negative (no = 0), or unclear (N/A = 0), and 10 items for assessing the quality of qualitative studies, each of which is scored as positive (yes = 2), partial (=1), or negative (no = 0). The quality assessment score is calculated as follows: the sum of each item score/28 for the quantitative studies, and the sum of each item score/20 for the qualitative ones, for a maximum quality score of 1.

## 3. Results

The main results are synthesized in Table 1. The following sections organize the results. First, we describe the sample included in the review and summarize the ATs described in the literature according to their characteristics. Then, the methods evaluating ATs users’ acceptance are reported by sorting them into quantitative and qualitative measures. Finally, we summarize the main outcomes emerging from the reviewed ATs encountered in the literature review.

### 3.1. Research Quality Assessment

Overall, the quality assessment score of the reviewed papers corresponded to a median score of 0.50 (averaged score, 0.51 and standard deviation = 0.13) [19]. The min score was 0.35 [20], and the max score was 0.8 [21,22]. The papers scoring over the median were 56% of the total [20,21,22,23,24,25,26,27,28,29] (See Table 1).

### 3.2. Sample Characteristics

In total, 532 participants were retrieved from the examined studies. Specifically, 185 participants had SCI [20,22,23,25,26,27,30,31,32,33,34,35,36,37]. Among those, 46 were people with tetraplegia (injury at C level), and 71 with paraplegia (damage at D and T levels), while for 68 of them, no information was reported about the level of disruption. One hundred sixty-four participants were reported to have acquired brain injury (ABI): 2 persons with ABI from post-stroke locked-in syndrome [32], 2 persons with ABI with no other specifications [32], 28 persons with stroke with no further specifications [21,27,38] 43 with ABI from ischemic stroke [24,39], 35 with ABI from haemorrhagic stroke [20,24,39], and 2 persons with hemiplegia with no other specification [20], 52 participants that were included in this group but with no further specification [24,27]. Thirty-seven participants were affected by congenital disorders: 1 by antropogirosis [32], 1 by spinal muscular atrophy [32] and 35 by cerebral palsy [27,32]. Thirteen participants were reported to have been subjected to amputations but without other specifications [27], plus another one with quadruple amputation [32]. One hundred thirty-three participants had acquired diseases: 83 were affected by multiple sclerosis [21,27,37], 12 by arthritis [27], 5 by polio [27], 8 with motor neuron disease and 25 by muscular dystrophy [27].

**Table 1:** Study characteristics.

Study	Sample (N)	Type of Injury	Type of Assistive Technology: Allocentric vs. Egocentric	Measures	Outcomes	Quality Assessment
[30]	9	Spinal cord injury	Egocentric—Hand exoskeleton (HX).	Ah-hoc test: comfort, safety, ease to use (6-point Likert).	Comfort (M = 4.17 SD = 4.92) Safety (M = 4.35 SD = 1.38) Ease of use (M = 3.12 SD = 1.81). Qualitative: modify the size and weight.	0.53
[31]	19	Spinal cord injury	Egocentric—REX walking aid.	Ad-hoc test: comfort, safety, ease to use, engagement (7-point Likert).	Total: M = 5.86 SD = 1.20	0.42
[32]	29	Tetraplegic: Spinal cord injury (*n* = 23), stroke (*n* = 2), cerebral palsy (*n* = 1), arthrogryposis (*n* = 1), quadruple amputee (*n* = 1), spinal muscular atrophy (*n* = 1).	Allocentric—Synthetic autonomous Majordomo robot (SAM).	Ad-hoc test: satisfaction, learnability, confidence in the system, ease to use (5-point Likert).	Satisfaction (M = 3.97 SD = 1.03), learnability (M = 4.21 SD = 0.7), Trust (M = 3.31 SD = 1.69), ease to use (M = 4.62 SD = 0.3).	0.5
[27]	262	* Multiple sclerosis (*n* = 73), cerebral palsy (*n* = 37), and spinal cord injury (*n* = 31)	Allocentric—Electrically powered wheelchairs (EPWs).	Ad-hoc test: usability, safety, reliability, and satisfaction (5-point Likert).	Usability: (M = 4.16 SD = 0.95), safety (M = 4.3 SD = 0.9), reliability (M = 4.3 SD = 0.8), and overall satisfaction (M = 4.2 SD = 0.8).	0.5
[20]	13	Stroke (*n* = 7), spinal cord injury (*n* = 6)	Egocentric—mobile, patient-adapted, robot-assisted gait rehabilitation system (MOPASS).	SUS scale, Ad-hoc test: acceptance (score 0–100).	Usability: M = 54	0.35
[33]	17	Spinal cord injury	Allocentric—SAM.	Ad-hoc test: usability, (a) success rate for achieving the task, (b) success rate for completing the task without mistakes, (c) Success rate for completing the task on time (score 0–100).	Usability: M = 82. 94.1% were satisfied with the control mode, 76.5% were confident, 70.6% were interest in using it at home.	0.4
[34]	14	Spinal cord injury	Egocentric—EKSO or OEAGT robotic exoskeleton.	Ad-hoc test: satisfaction, learnability, usefulness, safety, and motivation to use (score 0–100).	Satisfaction (M = 81.2 SD = 20.1), learnability (M = 76,61 SD = 19.07), usefulness (M = 94.3 SD = 27.93); safety (M = 26 SD = 40.9); motivation (M = 91.2 SD= 15.6).	0.4
[39]	46	Stroke	EKSO or OEAGT.	Technology acceptance model test (7-point Likert).	Total: M = 4.85 SD = 1.71	0.42
[26]	6	Spinal cord injury	Allocentric—Adaptive head motion control for user-friendly support (AMiCUS).	Ad-hoc test: ease to use (5-point Likert).	Total: M = 4.21 SD = 0.91	0.42
[23]	6	Spinal cord injury	Allocentric—ASIBOT.	Quebec user evaluation of satisfaction test (5-point Likert)	Average for drinking action 0.91, brushing teeth 0.49.	0.5
[22]	28	Spinal cord injury	Allocentric—Robotic locomotor exoskeleton.	Focus group: experiences and perspectives, benefits and barriers, concerns and limitations, and suggestions.	Robotic exoskeletons were useful in therapy settings but not for daily life activities. Dissatisfaction with the devices due to an inability to use them in autonomy and safely.	0.8
[21]	20	Stroke (*n* = 10), multiple sclerosis (*n* = 10)	Egocentric—SEM™ Glove.	Semi-structured interview: usability.	Difficult to use due to the complexity of everyday life where a single activity may involve grasp, grip, and pinch. Limited ability to coordinate finger movements.	0.8
[38]	1	Stroke	Egocentric—Proof-of-concept glove.	Semi-structured interview: usability, acceptability, satisfaction.	Patients are motivated to use for training purpose, but they feel quite uncomfortable. The grasp and velocity must be adjustable to be appropriate for each patient’s needs.	0.7
[37]	7	Spinal cord injury (*n* = 4), Multiple sclerosis (*n* = 3).	Egocentric—REX walking aid.	Ad-hoc test: satisfaction (5-point Likert).	High satisfaction with ease of transferring in and out of the REX (M = 1.86; SD = 1.46) and with itsappearance (M = 1.83; SD = 0.98). Low satisfied with the ability to carry an item while using the Ekso (M = 4; SD = 0.71), but more satisfied with its transportability (M = 2.8; SD = 0.84).	0.5
[25]	1	Spinal cord injury	EKSO or OEAGT robotic exoskeleton.	Quebec user evaluation of satisfaction test (5-point Likert—adaptation)	Total: M = 3.8; SD = 1	0.4
[35]	21	Spinal cord injury	Egocentric—H2 exoskeleton.	Ad-hoc test: comfort, fatigue, enjoyment, motivation.	Median results: comfort = 6, fatigue = 3, enjoyment = 6, advantages = 5, motivation = 6	0.42
[24]	37	Stroke	EKSO or OEAGT robotic exoskeleton.	Intrinsic motivation inventory (IMI) (7-point Likert), credibility/expectancy questionnaire (CEQ) (score 0–100)	IMI (M = 74% SD = 17.16%), CEQ (M = 75% SD = 18.5).	0.5
[36]	5	Spinal cord injury	Egocentric—driven gait orthosis (DGO).	Intrinsic motivation inventory (IMI) (7-point Likert), credibility/expectancy questionnaire (CEQ) (score 0–100) (score 0–100).	IMI (M = 65% SD = 3.25%), CEQ (M = 60.7% SD = 20.6).	0.53

* Note: Largest diagnostic groups.

### 3.3. Assistive Technologies Characteristics

The articles included in the review have been sorted into two ad-hoc developed groups. The groups were called egocentric and allocentric according to the levels of assistance the ATs provided.

Egocentric ATs refer to devices that the users can wear to replace the limbs (totally or partially). Allocentric ATs refer to devices that work without involving the user’s body, such as those controlled by joysticks [7] (Table 1).

#### Assistive Technologies Testing Setting

Fifteen articles included in the review are proof of concept studies. Namely, participants use the ATs only once at the laboratory and are invited to answer the user test questionnaires. The other three studies included in the review tested the prolonged use of ATs; for example, Dolan and colleagues [27] included 262 habitual users of powered wheelchairs (the mean durations of wheelchair use were 13.3 years) and studied their experience. In particular, the researcher contacted the users and invited them to complete the usability questionnaires regarding their experience with the powered wheelchair.

Gagnon and colleagues [34] tested a powered exoskeleton for 18 training sessions in a laboratory setting. Each session lasted around 60 min/session from 6 to 8 weeks, with a frequency of 2–3 training sessions per week. Palmcrantz and colleagues [21] instructed their participants to use the glove in daily activities for 6 weeks at home.

### 3.4. Method to Evaluate Users’ Acceptance

**Figure 2:**
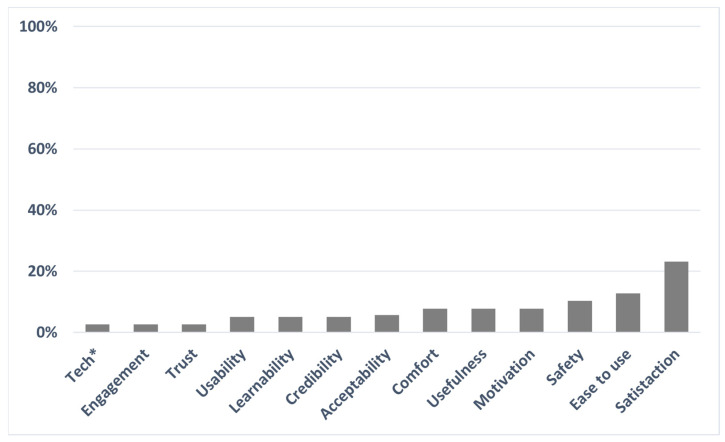
The user acceptance constructs used by the authors included in the review. The percentages indicate how many times each construct was administered. Note. * The tech construct refers to the technical aspects of the AT (i.e., weight, dimension).

To assess ATs’ end-user acceptance, three studies adopted qualitative measures (two semi-structured interviews and one focus group), while fifteen studies adopted quantitative measures with self-report tests (see Table 1). Figure 2 illustrates the constructs measuring participants’ acceptance. The measures include behavioural and technological constructs. The behavioural constructs measure the extent to which users control their behaviours entirely, in other words, the extent to which the behaviour is truly at the user’s discretion, such as satisfaction and motivation. The technological constructs measure characteristics related to technical aspects (e.g., height, weight, sound), usability, or comfort [40].

#### 3.4.1. Quantitative Measures

From the analysis, it emerged that the authors adopted ad-hoc or standardized questionnaires. All the ad-hoc questionnaires are reported in Table 2, and the standardized questionnaires are described below.

The Intrinsic motivation inventory (IMI) [41] was adopted in two studies [24,36]. IMI is a multidimensional questionnaire to assess participants’ experiences related to a target activity and consists of six subscales: Interest/Enjoyment, Perceived Competence, Effort/Importance, Pressure/Tension, Value/ Usefulness, and Relatedness. Items are rated on a 7-point Likert scale (1 “not at all true” to 7 “very true”). Higher scores indicate higher internal motivation, except for the pressure/tension subscale, where higher scores indicate greater feelings of pressure, which is conceived as a negative predictor of intrinsic motivation.

The credibility/expectancy questionnaire (CEQ) [28] was adopted in two studies [24,36]. It is a questionnaire to assess participants’ treatment expectancy and credibility, and it consists of two subscales: Credibility and Expectancy. Items are rated on a 9-point (1 “not at all” to 9 “very”) or an 11-point Likert scale (0% to 100%). Higher scores indicate higher credibility and expectancy.

The system usability scale (SUS) [28] was adopted in one study [20]. The questionnaire grounds the idea of presence as ‘being there’, that is, the illusion of feeling present in a certain scenario. It is composed of five questions related to three main issues: (i) the sense of being in the pit room, (ii) the extent to which this became the dominant reality, and (iii) the extent to which the experience was remembered as somewhere visited rather than just images seen. The questionnaire ranges from 0 (worst imaginable device) to 100 (best imaginable device).

The technology acceptance model scale (TAM) was adopted in one study [39]. The questionnaire was adapted from the original [42], and consists of seven subscales: comfortable, painful, strenuous, enjoyable, perceived usefulness, intention to use, and recommended. Items are rated on a 7-point Likert scale (1 “very unlikely” to 7 “very likely”).

The Quebec user evaluation of satisfaction with assistive technology [43] was adopted in two studies [23,25]. It is a questionnaire to evaluate a person’s satisfaction with a wide range of assistive technology. It is composed of six items rated on a 5-point Likert scale (1 “not satisfied at all” to 5 “very satisfied”).

**Table 2:** Ad-hoc items developed by the authors to measure user acceptance.

Construct	Items	Reference
Comfort	Do you feel comfortable with the orthosis? Is the process of preparation comfortable for you? Do you feel comfortable with the forearm fixations? Do you feel comfortable with the hand part of the orthosis? Do you think you can move your arm in a natural way while wearing the orthosis? Do you feel comfortable with the lateral pinch movement? Do you feel comfortable with the Palmar grasp movement? Do you feel comfortable with the precision grip movement? Do you feel comfortable with the power grip movement? Do you feel comfortable with the finger press and slide movement?	[30]
	I found REX comfortable	[31]
Safety	Do you feel safe with the orthosis?	[30]
	I felt very safe in REX I felt very confident in REX I felt very stable in REX	[31]
	I feel safe using my control device My control device is reliable	[27]
	I was afraid that the exoskeleton would have a mechanical breakdown I was fearful of losing my balance or falling I was afraid of causing or exacerbating musculoskeletal pains in my arms (wrists, elbows, shoulders) I was fearful of causing or exacerbating pains in my back around the level of my spinal cord injury and/or my spinal surgery I was afraid of causing or exacerbating neuropathic or neurogenic pains in my trunk or legs I was fearful of developing skin lesion(s) (wounds) due to pressure points or from the rubbing at different spots I was afraid that my blood pressure would drop	[34]
Ease to use	Do you think you can move your arm in a natural way while wearing the orthosis? Do you feel comfortable with the lateral pinch movement? Has been easy to learn how to use it? Would you use the orthosis in your daily live activities? Do you feel that the orthosis has improved?	[30]
	I found it easy to transfer into REX REX was easy to control	[31]
	Was use of the system fatiguing?	[32]
	My control device is easy to set up each time I use it My control device is easy to position where I need it to be My control device is easy to use	[27]
	It is easy to perform the gesture nodding down correctly It is easy to perform the gesture nodding up correctly It is easy to perform the gesture bending right correctly It is easy to perform the gesture bending left correctly Switching between the control groups is fast Switching between the control groups is easy	[26]
Engagement	I enjoyed my experience in REX I would like to use REX on a weekly I would recommend REX to a friend I felt a sense of wellness after using REX (mentally or physically) REX exceeded my expectations	[31]
Size, sound, speed	The size of REX did not bother me The sound of REX did not bother me The speed of REX was suitable for me	[31]
Satisfaction	I can see the benefits of using REX regularly I would like to see REX more accessible to those who need it	[31]
	Were you satisfied with the procedure for selecting the object’s location? Were you satisfied with the time taken by the mobile base to move to the object’s location? Were you satisfied with procedure for selecting the object to be grasped? Were you satisfied with how the object was grasped? Were you satisfied with the base’s return? Were you satisfied with the wide-angle visual feedback from the camera? Were you satisfied with the visual feedback from the webcams? Were you satisfied with how the mobile base’s movement was displayed on the screen? Were you satisfied with the interface (everything displayed in the screen)? Were you satisfied with use of SAM? (General satisfaction) Do you think that this robot could have practical uses at home (in your activities of daily living)? (General satisfaction) Do you think that use of SAM would reduce the amount of preparation or intervention by a carer at your home? (General satisfaction) If the system became commercially available, do you think you would purchase one for your home (if cost was not an issue)? (General satisfaction)	[32]
	Overall, I am satisfied with my control device	[27]
	The general appearance of the robotic exoskeleton is pleasing It is easy to transfer between my wheelchair and the robotic exoskeleton The robotic exoskeleton is easy to put on and off with the help of a therapist Wearing the robotic exoskeleton is comfortable The weight of the robotic exoskeleton has no effect on my walking The walking pattern generated by the robotic exoskeleton is similar to a normal walking pattern	[34]
	The dimensions (size, height, length, width) of the device? The weight of the device? The ease in adjusting (fixing, fastening) the parts of the device? How safe and secure the device is? The durability (endurance, resistance to wear) of the device How easy is it to use the device? How comfortable the device is? How effective the device is to solve the problem for which you are using it? I had no breathing difficulties while using the system After the experiment, I feel comfortable using the system	[25]
	How likely do you believe using the exoskeleton will help with the following? (from psychical to psychological benefit) How satisfied were you with the following while using the exoskeleton? (daily activiets) How necessary do you believe it is for the following features to be improved/further developed? (features of technology) If the exoskeleton were available for use, how likely would you be to: (home vs. community)	[37]
Learnability	How easy was it to learn to handle SAM?	[32]
	It is easy to learn to walk with the robotic exoskeleton I am satisfied with the progression over time of my skill level when walking with the robotic exoskeleton during the training sessions The sounds emitted by the robotic exoskeleton help me to rise from sitting and to sit down The sounds emitted by the exoskeleton help me while walking The instructions and feedback provided by the therapists helped my learning process to walk and do transitions (sit-to-stand and stand-to-sit) Watching short video clips of myself walking helped (or would have helped) my learning process for walking with the robotic exoskeleton I am satisfied with the skill level that I attained with respect to transitions between standing and sitting (sit-to-stand and stand-to-sit) with the robotic exoskeleton by the end of the training program I am satisfied with the skill level that I attained with respect to walking with the robotic exoskeleton by the end of the training program At the end of the training program, I feel that I could have walked without the help of a therapist considering my skill level with the robotic exoskeleton	[34]
Trust	Would you trust SAM to grasp fragile objects?	[32]
Usefulness (Perceived health benefits)	At the end of the training program, I noticed a general improvement in the state of my health At the end of the training program, I noticed an improvement in the muscular strength of my arms At the end of the training program, I noticed an improvement in the control of my sitting balance At the end of the training program, I noticed an improvement in general endurance for my daily activities At the end of the training program, I noticed the spasticity in my legs was decreased At the end of the training program, I noticed a reduction in the time required for moving my bowels At the end of the training program, I noticed an improvement in my sleep At the end of the training program, I noticed an improvement in my mental health and in my psychological well-being	[34]
	The graphical user interface is visually appealing and clearly arranged All necessary information is shown The feedback on the current head orientation is easy to understand and useful The gripper camera is useful The feedback about the gesture performance is useful	[26]
Motivation	At the end of the walking training program with the robotic exoskeleton, I felt motivated to take part in a physical activity program adapted to my condition At the end of the walking training program with the robotic exoskeleton, I plan to or I have continued to participate in moderate-to-strenuous physical exercise for at least 20 min twice weekly	[34]
Others ad-hoc measures		
Success rate for achieving and completing the tasks through the robotic arm, Acceptability: (1) Ergonomics of the system (command mode, learning, global use of the system) (2) Confidence in the SAM robot and accuracy of the object pick-up (3) Simplicity of the pick-up (4) Time required to execute the task.		[33]
Did you feel comfortable with the robotic therapy? (Comfort) Do you agree with the statement that you did not experience pain during the robotic therapy? (Absence of pain) Did you get tired during the robotic therapy? (Fatigue) Did you enjoy doing therapy with the robot? (Enjoy) Do you believe robot therapy sessions were beneficial? (Advantages) Would you like to perform more therapy with the robot? (Desire to continue) Would you suggest to perform therapy with the robot to anyone who suffered from stroke? (Suggest to anyone)		[35]

#### 3.4.2. Qualitative Measures

Structured interviews [21,38] and focus groups [22] were also adopted to investigate the acceptance of the ATs qualitatively. The structured interviews were focused on usability, acceptability, satisfaction, suggestions for improvement, and general comments [21]. On the other hand, the focus groups explored the patients’ experience with the robotic exoskeletons guided by questions such as “Tell me about your experience using a robotic exoskeleton”, “How have your previous experiences with technology influenced your thoughts on exoskeletons? [22]”.

### 3.5. Main Constructs Outcomes

#### 3.5.1. Satisfaction

Participants demonstrated overall satisfaction with the ATs. Regarding the allocentric devices, the hand robot of Coignard and colleagues [32] proved to derive an appreciation of the selection of the object’s different modes, in particular, the reaching and grasping mode by which one can interact with objects present in the environment, even if they are far from the user. In the study by Jardon and colleagues [23], which also tested a hand robot (ASIBOT), different responses emerged according to participants’ level of spinal cord injuries, i.e., participants with lesions between C3 and C6 were more satisfied with using the hand robot for drinking. In contrast, participants with lesions between C7 and C8 were less satisfied with the hand robot. For brushing teeth, participants with lesions between C4 and C6 were more satisfied than those with lesions between C5 and C8. Furthermore, all the participants were dissatisfied with the support in washing their faces [27]. Regarding the powered wheelchair, participants were more satisfied with using the control device of the wheelchair by hand than by chin or foot [27].

Regarding the egocentric devices, for the lower limb exoskeleton specifically, participants were shown to be satisfied with the locomotor training program (18 sessions for 6–8 weeks) through the robotic exoskeleton. However, they were slightly dissatisfied due to a lack of opportunity to continue the training with the robotic exoskeleton upon completion of the trial program [34]. Additionally, a stroke participant who tested the lower limb exoskeleton had an average of 3.8 out of 5 on the QUEST questionnaire of satisfaction [25]. Poritz and colleagues [37] tested two types of lower limb exoskeleton, REX and EKSO, and found general satisfaction with the amount of physical energy needed to stand and walk with the devices. However, participants reported being somewhat dissatisfied with the ability to walk over uneven surfaces and up and down stairs with either of the devices. Moreover, some differences in user satisfaction were observed between the REX and EKSO. Participants were more satisfied with the ease of transferring in and out of the REX and its appearance. Additionally, they were less satisfied with the ability to carry an item while using the EKSO, but more content with its transportability. Furthermore, participants reported that they liked the REX for the balance activity and the EKSO for the gait. However, they disliked the size and form of both REX and EKSO, the slow speed of the REX, and the feeling of instability when using the EKSO.

#### 3.5.2. Ease to Use

About the allocentric ATs, the HX by Almenara and colleagues [30] was found to be easy to use inside the laboratory, where it is possible to control the environmental features. On the contrary, they did not recommend using the device for daily activity in a domestic environment without an additional development stage since it was too complex to be used outside laboratory settings. The Majordomo robot [32] demonstrated that it had not exhausted its use in reaching objects, and participants were also confident in grasping fragile objects. The same results were obtained with the SAM robot [33].

The EPW showed that most end-users drive their wheelchair by hand, and only a few participants in the review drive theirs by the chin, switches, or foot. Furthermore, all participants considered the EPW easy to use. In particular, it emerged from the qualitative analysis that, for the participants using their hand, the joystick of the wheelchair was very intuitive and gave autonomy to the users (i.e., “It means I can be very independent”; “I can move about freely without relying on someone else as if I was in my manual chair all the time” (Participant) [27].

The AMICUS robot [26], which had the goal of selecting objects based on its following of the user’s head movements, was challenging for grasping, while moving the robot arm in the horizontal plane was rated as being more complicated. Gripper rotation (i.e., grasping the object from the table and rotating it) was assessed as the most challenging movement of the robot.

The ASIBOT [23], developed for tetraplegic users for ADLs, was easier to use with the tactile input, followed by voice recognition, the joystick and the lighted sequence last.

According to the egocentric ATs, for the REX exoskeleton, only 4 participants out of 20, could use the exoskeleton without any assistance (mostly paraplegics with cord injury levels between T1 and L5 and incomplete spinal cord lesions ASIA B-D); the others, primarily tetraplegic participants with cord injury levels between C4 and C8 or complete lesions ASIA A, needed assistance, such as a hoist [31], and sometimes the upright position was not reached, even with assistance. Moreover, the participants with severe spinal deformities lacked adequate trunk control to position the right upper limb to use the joystick and autonomously control the exoskeleton. In another study, most respondents agreed that it was easy to perform a sitting pivot transfer between their wheelchair and the robotic exoskeleton, and it was easy to put on and take off [34].

As for the MOPASS exoskeleton, the younger participants perceived the system as easier to use than the older ones [20]. Furthermore, younger participants reported that the system could be suitable for more severely affected people; on the other side, the older participants were worried about technical support.

The SEM glove, on the contrary, was found to be too bulky and heavy and too complicated to handle while dressing, moving, and transferring from a wheelchair, e.g., while using the toilet [21]. Moreover, even if the glove could help users to bring objects, such as a glass of water, the user could still not perform the activity due to the proximal weakness of the wrist or arm [21].

#### 3.5.3. Comfort

The comfort construct emerged only in the studies that mainly investigated the usability of the exoskeleton because users must wear the device. The HX of Almenara and colleagues [30] resulted in being slightly uncomfortable because it cannot adapt to different users’ hand sizes, being of one standard size only. Additionally, a lower limb exoskeleton, such as the EKSO, was comfortable to wear [24,34], whereas the MOPASS was more comfortable for older than for younger participants [20]. The H2 exoskeleton for gait rehabilitation scored only 3 out of 5 due to the participants’ low levels of control experienced during the experiment [25].

#### 3.5.4. Safety

Regarding physical safety, five studies examined the construct in relation to the exoskeleton. Participants using the REX reported no adverse events, such as signs of redness or bruising on their skin, and there were no cases of late notification of any adverse events [31]. From the qualitative results, we found that the MOPASS was perceived to be safer for older than for younger participants (i.e., “It helps me to walk safely without insecurities”) [20]. EKSO was also demonstrated to be a safe AT, that is, participants reported that walking with the robotic exoskeleton did not cause or exacerbate musculoskeletal pains in their wrists, elbows, shoulders, or pain in their back around the level of their spinal cord injury and/or spinal surgery. Moreover, it generated neither neuropathic nor neurogenic pains in their trunk or legs [24,25,34]. Participants also expressed no fear of the development of skin lesions, loss of balance or experience of blood pressure drops that are associated with walking by EKSO [34]. Similarly, the H2 exoskeleton was perceived to be safe only by participants who used it during the experiment [35].

#### 3.5.5. Learnability

Only three studies investigated the learnability of ATs. In particular, the ease of learning of MOPASS was rated as “very good” and “rather good” by younger and older participants, although two younger patients rated it as only “moderate” [20]. The SAM robot was easy to learn to reach objects. The same results were found for the EKSO exoskeleton [34]: most respondents agreed it was easy to learn to walk with the robotic exoskeleton. However, participants confirmed that the feedback they received from the certified therapists crucially facilitated learning. Furthermore, the different auditory feedback (i.e., sounds) emitted by the exoskeleton during sit–stand transfers and walking, together with the visualization of users’ performances captured via short videos and proposed through mobile phones (visual feedback), represented vital elements in developing motor control strategies [34]. However, Kinnett-Hopkins and colleagues [22] reported that participants found learning to use the lower limb exoskeleton challenging and that this was related to the first use.

#### 3.5.6. Usefulness

Palmcrantz and colleagues [21] underlined the usefulness of the SEM glove. It enabled users with hand impairments to produce more robust and sustained grips, as well as improving the grip precision, which allowed for the handling and holding of household objects (“The benefit has been that I have been able to use my left hand to lift things up and grasp things” (participant)). Moreover, the SEM was reported to enable the use of the gloved hand as an assistive hand while handling and holding household objects or when carrying objects in one hand while walking with assistive devices and pursuing bimanual activities, such as vacuum cleaning or pulling up trousers (“Really, I’m not strong enough to hold anything for a particularly long time…but, when I have had the glove, I have been able to hold a pack of butter and carry it to the table” (participant)) [21]. In the same study, the authors found that the water-proof characteristic is important for the perceived usefulness because they permit some daily activities such as handwashing and other self-care activities. However, if the users wear the SEM, they cannot use an iPad or smartphone unless they lift away the glove’s fingertips. Furthermore, the SEM was not assistive because it cannot grasp small and tiny objects, such as a knife and fork, a plate, the handle of a jug, a cup, or buttons [21]. Usefulness limits were also found with the proof-of-concept of Park and colleagues [38] for a glove, as the device’s velocity was not adjusted according to the user’s condition. On the contrary, the glove was appropriate for users with mild ability to open the hand and grasp the device.

#### 3.5.7. Motivation

Two studies investigated the motivation to use ATs. According to the IMI questionnaire’s results of Goffredo and colleagues [39], users showed high scores on intrinsic motivation to use the DGO exoskeleton, with a median ranging from 14 for pressure/tension to 45 for value/usefulness. However, the results of Jardon and colleagues [23] show that intrinsic motivation depends on the injury’s stage, that is, that users with the highest lesion level (C5) and with the longest time since injury are less motivated because they do not expect significant motor improvements.

## 4. Discussion

The present systematic review evaluated the acceptance of ATs of patients living with motor impairments due to spinal cord or acquired brain injuries.

We identified 18 relevant papers investigating the acceptance of 15 types of ATs. The participants included in the review were mainly people affected by SCI, with either tetraplegia or paraplegia, followed by patients who suffered from brain injuries and those with motor deficits with other aetiology.

The prevalence of SCI over ABI patients in the results may be related to the good cognitive abilities that allow participants to report their opinions about the experience with the technology. People with SCI have motor problems without cognitive deficits. On the contrary, people suffering from ABI may manifest cognitive impairments, reducing the probability of including them in the studies.

Most participants did not have previous experience with ATs, except in a study with 262 typical users of the powered wheelchair [27], and a study with 14 patients with SCI who tested the EKSO exoskeleton for 18 sessions [34]. The ATs studied were both egocentric and allocentric, namely wearable, such as an exoskeleton, assistive robot, or powered wheelchair. Except for the latter [27], all the ATs included in the systematic review were proof of concept and mainly tested in a laboratory setting. Moreover, while considering the technology readiness level, they were only at level four (technology validated in the lab) up to nine (actual system proven in operational environment) levels [44]. Thus, future studies should expend some effort into investigating ATs acceptance longitudinally and daily to account for the ATs dropouts.

According to the assessment of user acceptance, the review showed the need for more standardized measures. In fact, the analysis showed nine different constructs (Figure 2) assessed by heterogenous questionnaires, emerging from only four standardized measures: the QUEST [23,25], the SUS [20], the IMI, and the CEQ [24,36]. Alternatively, ad-hoc tests or qualitative measures were often used. One possible explanation for such heterogeneous methodology is the characteristics of the acceptance construct. There is no shared definition of acceptance, but it is moderated by several factors, such as the individuals’ characteristics in terms of their impairments and the physical and social environment [14,45]. Following this view, Federici [14] proposed the assistive technology assessment process (ATA). ATA depicts a user-driven process through which the comprehensive adoption of clinical measures, functional analysis, and psycho-socio-environmental assessments facilitates the evaluation and selection of ATs. In specific contexts of use, user well-being is targeted by matching user’s needs and ATs functionality. ATA represents a match assessment among different aspects, places the user at the centre of the evaluation process, and acknowledges the environment as crucial for AT acceptance [9]. However, in the studies included in the review, only the user’s perspective was tested: no evaluations of psychical and social environments were considered. For example, from the evaluation of the HX technology for hand assistance, it emerged that participants considered the AT easy to use in a laboratory setting where the environment is more controlled. However, they did not recommend the device for daily activities in domestic environments [30]. Future studies should develop standardized acceptance questionnaires to increase the reliability of future research in the field.

Another significant result emerged: satisfaction with the technology resulted in being correlated with the level of the spinal lesion. For example, regarding the ASIBOT technology, participants with a low spinal lesion were more satisfied using the hand robot than those with a higher lesion [23]. Only participants with paraplegia and incomplete lesions (ASIA B-D) could use the REX exoskeleton without assistance. Participants with tetraplegia and complete spinal cord lesions (ASIA A) needed assistance, i.e., a hoist [31]. Instead, the MOPASS exoskeleton was easier to use for young participants than their older counterparts [20]. These results underline the difficulty of developing a standardized AT acceptable to many potential users and the need for tailored AT customizations [14].

In general, participants were satisfied with the offered ATs. They felt safe and experienced no adverse events. They found ATs to be comfortable and easy to learn. However, the results should be interpreted cautiously because the papers reported the outcomes of pilot studies conducted in laboratory settings. Future works should focus on ATs used during daily activities longitudinally and at users’ homes.

## 5. Critical Aspects

Even with the soundness of the present work procedure, some aspects need consideration.

Firstly, the included studies are limited to reporting significant conclusions. However, the limited number underlines the novelty of the field and the need to put more effort into investigating the role of acceptance toward ATs by people with motor impairments due to spinal cord or acquired brain injuries.

Second, the quality of the reviewed studies could have been better: only two out of eighteen studies presented a good quality average score (i.e., a score higher than the group mean score plus two standard deviations [21,38]). This may derive from the pilot nature of the studies, which were “proof-of-concept” regarding assistive technology: no randomized controlled trial procedures were used. According to the methodological research quality assessment [19], high-quality research is based on adequate sample sizes and the adoption of blind procedures was prevented in the reviewed studies that directly investigated participants’ opinions regarding specific technologies.

At last, even if the inclusion criteria limited the search to two aetiologies (SCI and ABI), a few papers also included persons manifesting motor problems derived from other aetiologies [21,27,32,37]. However, the results neither reported differences nor sorted the results according to patients with different aetiologies. This might not be surprising as, according to the global report on health equity for persons with disabilities (see introduction), AT is expected to match personal needs based on a specific functional limitation that can, however, result from completely different nosographies and aetiologies. However, in future studies, it might be important to better investigate whether aetiologies can play a role [46] and whether the profession of the specialists involved in the evaluation of the acceptance of assistive technologies may represent a source of bias.

## 6. Conclusions

In conclusion, the field of AT’s acceptance by patients with motor impairments due to spinal cord or acquired brain injuries needs more attention from the scientific community. Our search shows that this is the first systematic review in the field. Consequently, this underlines the limited attention it has received so far. This could be due to the lack of financial resources as users’ experience studies on ATs might not be a priority of the research entities’ funding. Furthermore, the studies on users’ experience need the collaboration of researchers with different profiles, such as biomedical engineers, healthcare personnel, and psychologists, meaning it may be a challenge to arrive at a shared conclusion. At last, recruiting people with motor impairments due to spinal cord or acquired brain injuries is difficult as they need special attention and, sometimes, the research centres are full of architectural barriers preventing potential participants from leading the study.

Despite the limitation of only finding and including short-term studies, the present review synthesized the main constructs and the respective items adopted by the authors to assess the acceptance of the technology. In particular, as we found a lack of standardized measures (i.e., questionnaires), we present a useful and comprehensive sample of ad-hoc items for future studies to investigate users’ acceptance in Table 2. This certainly represents a novel aspect offered by the present review.

To sum up, results emphasize the crucial role of the users in developing ATs that are as customizable as possible and capable of minimizing early abandonment risks. As has emerged from this review, the characteristics of a person with motor disability, those of the environment, caregivers included, and those of technology should be weighed thoroughly to select the most appropriate AT. Within the perspective represented by the human–technology interaction paradigm, the person pivots all the selection, adaptation, and assignment processes of a specific AT, which would secure high acceptance rates and low risk of abandonment.

## Data Availability

Data is available upon request.
